# Preoperative neutrophil to lymphocyte ratio (NLR), lymphocyte to monocyte ratio (LMR), and platelet to lymphocyte ratio (PLR) as prognostic markers in patients with retroperitoneal liposarcoma

**DOI:** 10.1016/j.sipas.2022.100076

**Published:** 2022-03-29

**Authors:** Dorian Yarih Garcia-Ortega, David Ponce-Herrera, Alethia Alvarez-Cano, Claudia Caro-Sanchez, Kuauhyama Luna-Ortiz

**Affiliations:** a Surgical Oncology, Skin and Soft Tissue Tumors Department, National Cancer Institute, Ave. San Fernando 22 Col. Seccion XVI Tlalpan, Mexico City 14080, Mexico; b Fellow Surgical Oncology, Surgical Department, National Cancer Institute, Mexico City, Mexico; c Surgical Oncology, Christus Muguerza Alta Especialidad, Monterrey, Nuevo Leon, Mexico; d Oncologic Pathology, National Cancer Institute, Mexico City, Mexico; e Surgical Oncology Department of Head and Neck Surgery Department, National Cancer Institute, Mexico City, Mexico

**Keywords:** Retroperitoneal liposarcoma, Neutrophil-to-lymphocyte ratio, Prognostic factor, Systemic inflammatory responses

## Abstract

Retroperitoneal liposarcomas are rare mesenchymal tumors of that are typically detected in advanced stages and often carry a poor prognosis. The recurrence rate is high even after an adequate treatment. The multimodality therapy is not a standard for every case; therefore, an individual risk assessment is needed to select tailored treatment plans. Several inflammatory ratios have been proposed as prognostic factors and may aid in the treatment selection.

**Objective:**

To analyze the impact of preoperative inflammatory-related ratios as prognostic factors in patients with retroperitonea liposarcoma.

**Methods:**

We retrospectively evaluated 87 individuals diagnosed with retroperitoneal liposarcoma from a high-volume sarcoma center during January 1, 2008, to December 31, 2018. The relation between preoperative inflammatory indices (neutrophil/lymphocyte, lymphocyte/monocyte and platelet/lymphocyte ratios) and the disease-free survival (DFS) and overall survival (OS) were analyzed.

**Results:**

Fifty (57.5%) participants were men and thirty-seven (42.5%) were women. The mean age at diagnosis was 53.64 years (SD ± 13.18). The mean tumor size was 27.79 cm (SD ± 13.48). The most common histological subtype was dedifferentiated liposarcoma (ddLPS) in 49.4% (*n* = 43) cases, followed by well-differentiated liposarcomas (wdLPS) in 44.8% (*n* = 39) cases. An analysis of the ROC (receiver operating characteristic) curve. was applied, and only the NLR was useful and associated with a worse OS (*p* = 0.047), with a cut-off point of 2.77.

**Conclusion:**

In our series, the NLR > 2.77 was an independent prognostic factor associated with lower overall survival in our series. Due to its easy accessibility and reproducibility, we believe that it can be a useful test in the clinical practice and potentially be included in risk prediction nomograms.

## Background

Liposarcomas (LPS) are the most common soft-tissue sarcoma (STS) in adults. The World Health Organization divides LPS into four subtypes: atypical lipomatous tumor/well-differentiated liposarcoma (wdLPS), myxoid liposarcoma (mLPS), dedifferentiated liposarcoma (ddLPS), and pleomorphic liposarcoma (pLPS) [1]. The most frequent locations of LPS are the extremities, followed by the retroperitoneum where it is also the most frequent histological LPS subtype. This disease represents a challenge not only during the surgical management, but additional treatment modalities have little benefit [2,3]. A complete surgical resection, with negative margins, is pivotal for the curative-intent treatment. Retroperitoneal liposarcomas (rtpLPS) have shown a 5 year DFS and 5 year OS of 41–50% and 54–70%, respectively. However, around 50% of patients with adequate local control still develop distant metastases or local recurrence and eventually die from this disease [4], [5], [6], [7].

The tumor microenvironment and, in particular, the inflammatory response play an important role in the development and progression of cancer and may be associated with systemic inflammation; Measurable parameters in the blood that reflect the systemic inflammatory response are elevated C-reactive protein, hypoalbuminemia, elevated levels of some cytokines, and elevated levels of leukocytes and their subtypes. Immune cell components of the complete blood count (CBC) offer a particularly attractive measure of inflammation because the CBC is often collected as part of standard clinical care at minimal cost. An available marker of systemic inflammation of the CBD is the neutrophil-to-lymphocyte ratio (NLR); this is because neutrophilia is a common feature of chronic inflammation associated with cancer; and although neutrophils are an integral part of the innate immune response, immunosuppressive and tumor-promoting functions of some subpopulations of neutrophils have been documented. In addition to producing cytokines associated with tumor progression, neutrophils can suppress cytotoxic T cell activity and, in turn, promote metastasis. Neutrophilia is often accompanied by relative lymphocytopenia, which represents a significant decrease in the cell-mediated adaptive immune response. The NLR captures the balance between the deleterious effects of neutrophilia and the beneficial effects of lymphocyte-mediated adaptive immunity. Both systemic neutrophilia and lymphopenia are associated with a worse prognosis in cancer patients; a high proportion of RLNs before treatment is associated with poorer survival outcomes [8], [9], [10].

In the last decades, different biomarkers have been used to try to identify prognostic groups in cancer, STS are not the exception. Multiple studies suggest that the inflammatory response plays a decisive role in tumor development by promoting angiogenesis, tumor invasion, increasing the cellular metastatic hability, altering the innate and adaptive immune response, and increasing the survival and proliferation of tumor cells [11,12]. Several prognostic indices based on complete hemograms have been proposed, including the modified Glasgow prognostic index, neutrophil-lymphocyte ratio (NLR), platelet-lymphocyte ratio (PLR) and lymphocyte-monocyte ratio (LMR) [13], [14], [15], [16], [17], [18], [19], [20], [21].

However, there is no literature that focuses on the correlation between inflammatory markers and the prognosis of rtpLPS. The objective of this study was to analyze the relation between several proinflammatory rations and the oncologic outcomes after radical surgical treatment.

## Patients and methods

We retrospectively analyzed data collected from the medical files of 87 patients with rtpLPS treated in a single high-volume sarcoma center, between January 2008 and December 2018. Clinical data were obtained from medical records and included age, sex, tumor size, admission status and treatment received. Following the WHO classification, the histological subtype was classified as wdLPS, ddLPS, mLPS/rLPS and pLPS. The multifocal disease was defined as having more than one distinct tumor nodule larger than 1 cm. The integrity of the tumor was classified as fragmentary (resection in parts or rupture of the tumor during resection) or complete resection based on the surgical report and / or the pathological report. Preoperative whole-blood cell counts were also analyzed.

The inclusion criteria were: individuals older than 18 years of age; diagnosis of rtpLPS histologically confirmed by two experienced pathologists, and radically resected localized disease (R0 / R1 resection).

The exclusion criteria were: metabolic conditions (uncontrolled diabetes mellitus defined as glucose >126 mg/dl fasting or >200 mg/dl), active respiratory, gastrointestinal or urinary tract infection that is or is not under antimicrobial management, chronic inflammatory or autoimmune diseases that are in active phase or with the use of immunosuppressants or immunomodulators (rheumatoid arthritis, systemic lupus erythematosus, etc.), being treated with chemotherapy or radiotherapy.

The NLR was obtained by dividing the neutrophil count by the lymphocyte count. The PLR was obtained by dividing the platelet count by the lymphocyte count. The MRL was obtained by dividing the lymphocyte count by the monocyte count, and the PMR was obtained by dividing the platelet count by the monocyte count.

After resection, all patients were scheduled for surveillance imaging: CT scans or MRI every 3,4 months during the first year, then every 6 months for the following 3 years, and then annually. The overall survival (OS) was defined as the time from diagnosis to the last follow-up or death. Disease-free survival (DFS) was defined as the time from the last treatment to clinical or radiological recurrence confirmed by histological analysis; the results are expressed in months. Recurrence was defined as clinical evidence (by physical examination and / or imaging) with histological confirmation by biopsy after 6 months. All patients were followed up until December 2018, the time of final follow-up or the date of death.

## Statistical analysis

The SPSS version 26 statistical program was used. A descriptive analysis of the variables was performed, as well as measurements of central tendency and dispersion according to their type of distribution. ROC curves were used to determine the cut-off point for the neutrophil-lymphocyte index and the rest of the inflammatory indices and thereby dichotomized the population. They were cataloged as high or low indices and were analyzed with X2. Student's *t* tests were applied for independent variables. Survival analyses were performed using log rank tests and Kaplan-Meier graph.

## Results

We reviewed a total of 103 medical files of individuals who had a diagnosis of retroperitoneal liposarcoma. Eighty-seven patients met the inclusion criteria, of whom 50 (57.5%) were men and 37 (42.5%) were women. The mean age at diagnosis was 53.64 years (SD ± 13.18). Table 1 shows the demographic characteristics of our cohort. Median follow up was 55 months

**Table 1:** Clinical and demographic characteristics of 87 patients with retroperitoneal liposarcomas.

	**TOTAL*N* = 87**	**INL <2.77*N* = 38**	**INL >2.77*N* = 49**	***p***
Age	53.64 (±13.18)	52.34 (±12.56)	54.65 (±13.69)	*0.421*
Sex Males Females	50 (57.5) 37 (42.5)	17 (44.7) 21 (55.3)	33 (67.3) 16 (32.7)	*0.034*
Tumor size	27.79 (±13.48)	25.22 (±12.64)	29.77 (±13.90)	*0.123*
Histological subtype WDLS DDLS PLS	39 (44.8%) 43 (49.4%) 5 (7.2%)	25 (65.8%) 11 (28.9%) 2 (5.3%)	14 (28.6%) 32 (65.3%) 3 (6.1%)	*0.003*
T^a^ T1 T2 T3 T4	0 7 (8.0%) 10 (11.5%) 70 (80.5%)	0 7 (18.4%) 4 (10.5%) 27 (71.1%)	0 0 6 (12.2%) 43 (87.8%)	*0.007*
N^b^ N0 N1	-	-	-	
M^c^ M0 M1	84 (96.6%) 3 (3.4%)	38 (100%) 0	46 (93.9%) 3 (6.1%)	*0.121*
G^d^ G1 G2 G3	32 (36.8%) 12 (13.8%) 43(49.4%)	22 (57.9%) 5 (13.5%) 11 (28.9%)	10 (20.4%) 7 (14.3%) 32 (65.3%)	*0.001*
Stage^e^ IA IB II IIIA IIIB IV	0 33 (37.9%) 0 1 (1.1%) 50 (57.5%) 3 (3.4%)	0 22 (57.9%) 0 1 (2.6%) 15 (39.5%) 0	0 11 (22.4%) 0 0 35 (71.4%) 3 (6.1%)	*0.002*
Recurrence No Yes	34 (39.1%) 53 (60.9%)	12 (31.6%) 26 (68.4%)	22 (39.1%) 27 (55.1%)	*0.207*
Death No Yes	26 (29.9%) 61 (70.1%)	16 (42.1%) 22 (57.9%)	10 (20.4%) 39 (79.6%)	*0.028*

a ^Primary Tumor.^

b ^Regional lymph nodes.^

c ^Metastasis.^

d ^Degrees based on the Federation Nationale des Centers de Lutte Contre le Cancer (FNCLCC) group.^

e ^Clinical stage based on the American Joint Committee on Cancer (AJCC) staging system.^

The mean tumor size was 27.79 cm (SD ± 13.48). The most common histological subtype was ddLPS in 49.4% (*n* = 43) of the cases, followed by wdLPS in 44.8% (*n* = 39). T4 grade was observed in 80.5% (*n* = 70) individuals. 50 patients (57.5%) were in clinical stage IIIB, 33 (37.9%) were in clinical stage IB, one patient (1.1%) was in clinical stage IIIA. No one was diagnosed in clinical stages IA or II.

Upon admission to the Institute, 60 patients were treatment-naive while 27 had gone through an unplanned surgery at a different center. After surgical treatment, only 30 patients received some type of adjuvant therapy. The main adjuvant treatment modality given was radiotherapy in 22 cases, 5 cases received chemotherapy and only 3 cases received both treatment modalities.

After follow up, 69.0% of patients (*n* = 53) presented recurrence (local or distant). The median DFS was 17 months (IQR 8–35). During the 60-month follow-up period, 70.1% of patients (*n* = 61) died, with a median OS of 36 months (IQR 17–60).

**Fig. 1:**
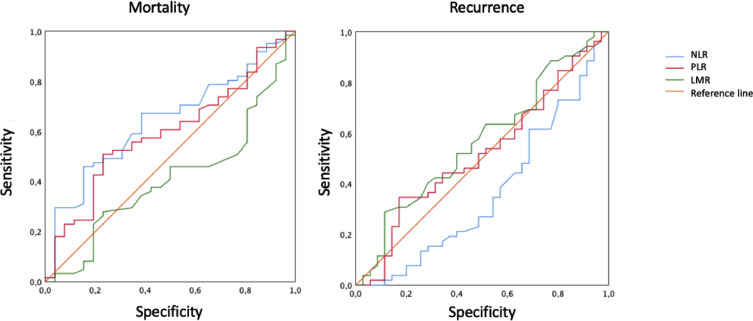
ROC curves for the 3 inflammatory indexes in mortality and recurrence.

**Fig. 2:**
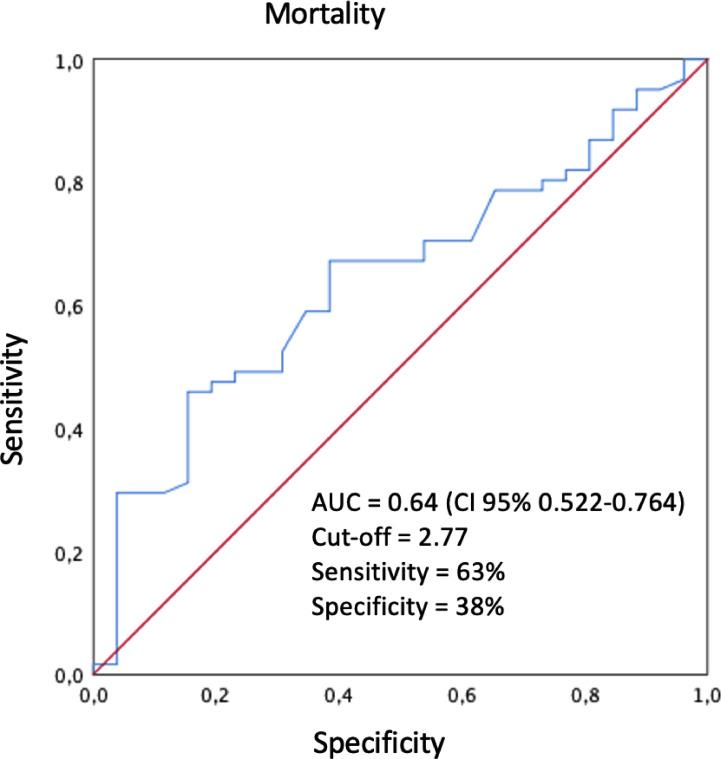
COR curves for INL in mortality. AUC = Area under the curve.

**Fig. 3:**
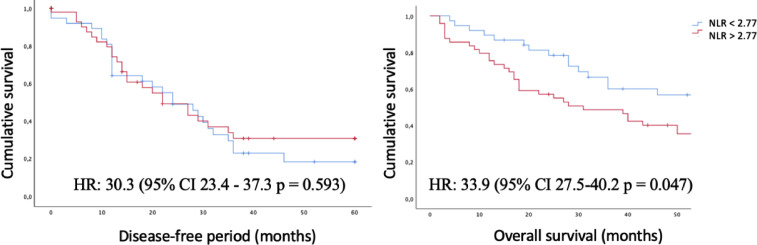
Disease-free period and overall survival by neutrophil-lymphocyte ratio.

Receptor operating curve (ROC) analyses were applied for the three inflammatory indices (Fig. 1), both for PLE and SG; only the NLR showed significance. The cut-off point was determined at 2.77. This was then used to analyze DFS and OS (Fig. 2, Fig. 3).

Statistical analyses of the Kaplan Meier curves were performed comparing the high or low NLR for both recurrence and mortality with a HR: 30.3 (95% CI 23.4–37.3 *p* = 0.593) and HR: 33.9 (95% CI 27.5–40.2 *p* = 0.047), respectively.

## Discussion

The medical management of sarcomas is a therapeutic challenge, as it requires an adequate evaluation and preoperative planning. The mainstay of treatment is surgery, for an adequate surgery highly impacts survival and recurrence. Negative margins and non-fragmentation of the surgical piece are the most important variables dependent on the surgeon. Multiple factors that impact on the evolution of the prognosis of the disease have been evaluated (beyond the maneuvers dependent on the surgeon), particularly the histological grade and clinical stage.

The use of inflammatory markers has been proposed as a prognostic factor for cancer outcomes. Due to the incidence of sarcomas, most of the series reported have included soft tissue sarcomas in different localization sites (extremities, trunk and retroperitoneum) and bone sarcomas. To our knowledge, this is the first study specifically conducted in patients with rtpLPS from a single high-volume center in Latin America. Up to today, there are only two specific studies of rtpLPS. In the study published by Luo et al. [22], no association between NLR and DFS/OS was found. On the other hand, Yali et al. [23] reported that elevated NLR did have an impact as a predictor of worse OS, which is similar to our series.

Our study population is very similar to other series, including the male:female ratio and a greater number of ddLPS than wdLPS. The average tumor size was larger than what has been reported globally. Several factors could be related to the high proportion of patients with high grade tumors, such as a delayed diagnosis and referrals [4,6,[24], [25], [26]]. Our study found that an initially elevated NLR is associated with poorer survival in patients undergoing surgical resection for rtpLPS. Other independent prognostic factors with statistical significance for mortality were histological grade, clinical stage, and histological subtype.

Currently, the trend is to use computer tools to help predict the prognosis of sarcomas, such is the case of SARCULATOR [27] that includes well-known prognostic variables in sarcomas; however, it does not yet include the use of inflammatory markers as part of its prognostic variables. Inflammatory markers can be a useful, simple, inexpensive, and easily reproducible tool that could be part of a nomogram to predict response, survival, and recurrence.

Finally, it is important to mention that our study has some limitations; first, it is a retrospective study of a single cancer center; second, the variety of situations upon admission to the institute (as the patients had previously received treatments); and third, the time window of the study, since during the development of the study an improvement in the rate of complete resections (R0-R1) and less incidence of tumor fragmentation was observed. Therefore, multicenter and prospective studies are required to better assess the sensitivity and specificity of the NLR as a prognostic predictor.

## Conclusion

There is a clear association in worse overall survival in patients with retroperitoneal liposarcoma who present a NLR> 2.77, but not for DFS. This is a simple, reproducible and inexpensive test that could be considered when making therapeutic decisions.

## Ethics approval

This research study was conducted retrospectively from clinical files and used for clinical purposes. An IRB official waiver of ethical approval was granted by the IRB of *National Cancer Institute* (Mexico).

## Funding

None.

## CRediT authorship contribution statement

**Dorian Yarih Garcia-Ortega:** Conceptualization, Resources, Data curation, Formal analysis, Writing – original draft, Writing – review & editing. **David Ponce-Herrera:** Conceptualization, Resources, Data curation, Formal analysis, Writing – original draft, Writing – review & editing. **Alethia Alvarez-Cano:** Conceptualization, Writing – review & editing. **Claudia Caro-Sanchez:** Conceptualization, Writing – review & editing. **Kuauhyama Luna-Ortiz:** Conceptualization, Writing – review & editing.

## Declaration of Competing Interest

The authors declare that they have no conflicts of interest.
